# MicroRNA-155 expression suggests a sex disparity in innate lymphoid cells at the single-cell level

**DOI:** 10.1038/s41423-019-0303-4

**Published:** 2019-10-10

**Authors:** Carina Malmhäll, Julie Weidner, Madeleine Rådinger

**Affiliations:** Krefting Research Centre, Sahlgrenska Academy, University of Gothenburg, Gothenburg, Sweden

**Keywords:** miRNA in immune cells, Innate lymphoid cells

MicroRNAs (miRNAs) are small noncoding RNAs that control gene expression at the post-transcriptional level, thereby serving as important cellular regulators.^1^ However, miRNA expression in specific human cellular subtypes, especially at the single-cell level, remains largely unexplored. In this study, a novel method called the PrimeFlow™ RNA Assay^2^ was used to directly monitor miRNA expression in the cell. Notably, we demonstrate for the first time that human innate lymphoid cells (ILCs) express miR-155. We further demonstrate a clear distinction between the sexes, with a significant increase in the number of ILCs expressing miR-155 in female-derived cells compared with male-derived cells upon in vitro stimulation.

Asthma is a chronic inflammatory disease of the airways that affects millions of people worldwide. Airway inflammation is triggered by allergens, viruses, and other particles, leading to the release of alarmins, which attract and activate inflammatory cells and release a cascade of inflammatory mediators. The recently discovered ILCs are potent drivers of inflammation and are considered the innate counterpart of T helper (Th) cells in the adaptive immune system.^3^ We have previously described the importance of miR-155 in both type 2 ILCs (ILC2s) and Th2 cells in murine models of asthma.^4,5^ miR-155 deficiency resulted in impaired interleukin-33 (IL-33) signaling and ILC2 function in response to both acute and chronic allergen exposure.^4^ Due to the rarity of ILCs, the most common experimental approach involves sorting and expanding these cells in vitro prior to experimental analyses. In this study, we explored the possibility of using a mixed-cell population and subsequently analyzed these rare cells without prior sorting. The PrimeFlow™ technique enables the simultaneous detection of a specific miRNA and antibody staining of cell surface markers, allowing for the quantitative detection of miRNAs at the single-cell level in heterogeneous cell populations. We hypothesized that the change in miR-155 expression in ILCs upon stimulation would be different in asthmatic patients than in healthy individuals. In addition, we hypothesized that sex-based differences might occur, since females have an increased risk of asthma compared with males.^6^ Therefore, the aim of this study was to assess miR-155 expression in ILCs, ILC2s, and Th cells at the single-cell level.

We recruited 13 well-characterized subjects, which included healthy controls and asthmatic subjects of both sexes, from the West Sweden Asthma Study (WSAS) cohort.^6^ All subjects gave written informed consent. Ethical approval for this study was granted by the Regional Ethical Approval Committee in Gothenburg, Sweden (no. 228-14). Blood samples were obtained, and peripheral blood mononuclear cells (PBMCs) were isolated and subsequently subjected to three stimuli, IL-33, IL-33+prostaglandin D2 (PGD2), or polyinosinic–polycytidylic acid (P(I:C)) for 22 hours. Both ILC2 and Th2 cells express the IL-33 receptor ST2 and/or the PGD2 receptor CRTH2.^3^ In addition to its role in ILC2 function, IL-33 is important in immune defense, serving as a guardian of barriers, and IL-33 was used to mimic the release of this alarmin from damaged epithelium.^7^ PGD2 induces cell migration, contributes to type 2 cytokine production, and is released from inflammatory cells, such as mast cells.^8^ In the third condition, a synthetic analog of double-stranded RNA, P(I:C), was used as a viral mimic. PBMCs were processed according to the manufacturer’s instructions throughout the staining and hybridization procedure (PrimeFlow™ RNA Assay with microRNA Pretreatment Protocol) by using a commercial assay kit (PrimeFlow™ RNA Assay, Invitrogen by Thermo Fisher Scientific, Affymetrix, Santa Clara, California). To identify the different cell subsets, cells were labeled with surface antibodies. Lineage-negative cells were defined as CD45^+^, CD3^−^, CD14^−^, CD16^−^, CD19^−^, CD20^−^, CD56^−^, CD123^−^, CD11c^−^, and FcεRI^−^. ILCs were defined as CD45^+^, Lin^–^, and CD127^+^. ILCs positive for CRTH2, ST2, or both markers were considered ILC2s. CD45^+^Lin^+^CD4^+^ cells were considered Th cells. For the detection of miR-155 expression, we used a single-pair target probe set for human miR-155-5p (MIMAT0000646, VM1-10254-PF, QuantiGeneView RNA miRNA Probe Set, Affymetrix Inc.). By using flow cytometry analysis, we evaluated the differences between asthmatic and healthy individuals in regard to the number of miR-155^+^ILCs, as well as the differences between sexes (Fig. 1a). No differences were found between healthy and asthmatic individuals, possibly because the individuals with asthma had mild-to-moderate asthma that was well-controlled. However, a clear distinction between the sexes was observed, with an increase in the number of ILCs expressing miR-155 in females compared with males (Fig. 1a).

**Fig. 1:**
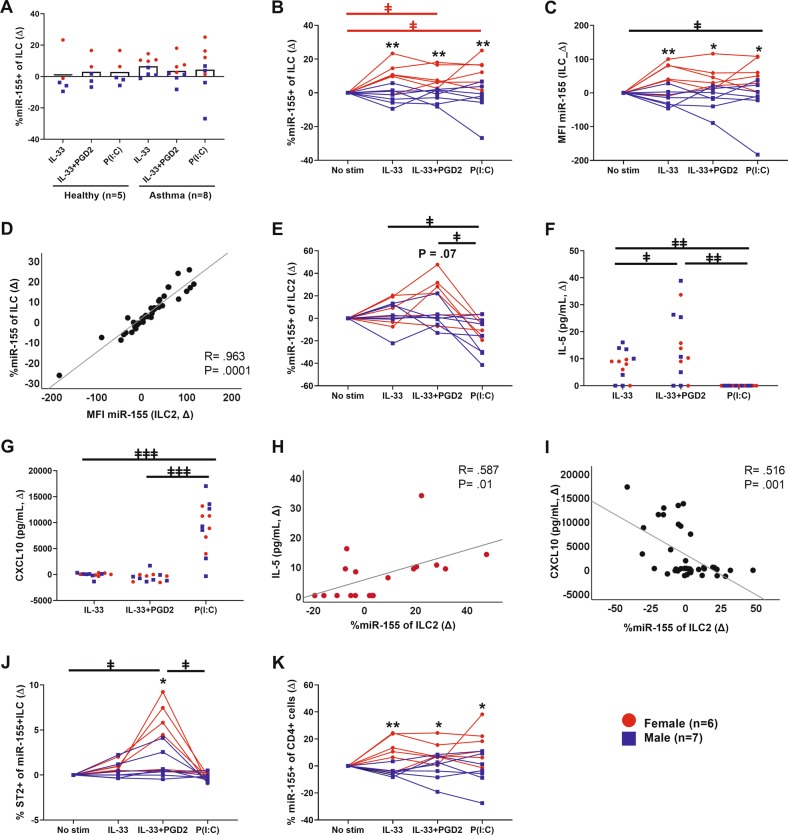
Sex disparity of miR-155 expression. PBMCs were seeded at a concentration of 2 × 10^6^ cells/ml and were cultured in RPMI 1640 media containing 1% L-glutamine, 1% penicillin–streptomycin (all from GE Healthcare Life Sciences HyClone Laboratories, Logan, Utah), 10% heat-inactivated AB+ human serum (in-house), and 100 U/ml recombinant IL-2 (BD Pharmingen™, BD Biosciences). Cells were subsequently subjected to three stimuli: 50 ng/ml recombinant IL-33 (PeproTech, Rocky Hill, New Jersey), a combination of 50 ng/ml IL-33+100 nM of prostaglandin D2 (PGD2; Cayman Chemical, Ann Arbor, Michigan), or 10 µg/ml P(I:C) (Poly(I:C) HMW VacciGrade™ InvivoGen, Toulouse, France) for 22 hours. This study was performed as five separate experiments. Upon the stimulation of cells, **a** there was no difference between healthy and asthmatic individuals in the numbers of miR-155^+^ILCs. There were sex differences in **b** the numbers of miR-155^+^ILCs, **c** and miR-155 expression in ILCs based on the median fluorescence index (MFI) values. Additionally, there were sex differences in the **j** number of miR-155^+^ILCs expressing ST2 and **k** the number of miR-155^+^CD4^+^ cells. **d** Correlation between the numbers of miR-155^+^ILCs and miR-155 expression in all subjects. **e** Numbers of miR-155^+^ILC2s. Release of **f** IL-5 protein and **g** CXCL10 protein in cell-free supernatants. Correlation between miR-155^+^ILC2s and **h** IL-5 in females and **i**) CXCL10 in all subjects. All data are expressed as changes from the no stimuli control (∆) for each individual. Female data are shown in red, male data are shown in blue, and data from all subjects are shown in black. *P < 0.05 and **P < 0.01 indicate differences between the sexes; ^*ǂ*^*P* < 0.05, ^ǂǂ^*P* < 0.01, and ^ǂǂǂ^*P* < 0.005 indicate differences between stimuli; significance is color-coded as previously described. Mann–Whitney tests were used for comparisons between females and males, and the Friedman test followed by Dunn’s multiple-comparison test was used for comparisons between stimuli. Correlations were performed by using Spearman’s rho, with R indicating the Spearman coefficient

The regulation of miRNA expression from a sex perspective has been described as being due to two main factors: the enrichment of miRNAs on the X chromosome and the estrogen regulation of miRNA transcription and processing.^9^ It is possible that the latter factor, but not the former factor, applies to miR-155, as miR-155 is found on chromosome 21q21. It was previously shown in murine models that uterine ILC2s express estrogen receptor, whereas lung ILC2s do not.^10^ Currently, it is unclear whether human blood-derived ILCs express estrogen receptors, and we could not determine whether the distinct miR-155 expression in ILCs between the sexes was directly regulated by sex hormones. Our results demonstrated that ILCs responded similarly to each stimulus, both in terms of the number of miR-155^+^ILCs and miR-155 expression per cell, which was evaluated based on the median fluorescence index values, which showed that females had ILCs that expressed more miR-155 than males (Fig. 1b, c). Furthermore, we observed a clear correlation between the cell number and the expression of miR-155 in all subjects (Fig. 1d). We next evaluated ILC2s, as these cells would likely respond directly to IL-33+PGD2. When these cells were stimulated with IL-33+PGD2, a trend was observed toward a difference between females and males (p = 0.07; Fig. 1e). The virus mimic P(I:C) did not induce miR-155 expression in ILC2s in either sex, suggesting that the miR-155 upregulation seen in total ILCs occurred in a different subtype and not in ILC2s. Next, we used ELISAs (Human IL-5 DuoSet, Human CXCL10/IP-10 DuoSet; R&DSystems, Minneapolis, Minnesota) to measure the release of mediators such as IL-5, a type 2 cytokine released from ILC2s, and CXCL10, a chemokine that is often measured to demonstrate that there is a type 1 response. IL-33 and the combination IL-33+PGD2 resulted in an increase of IL-5 (Fig. 1f), and P(I:C) resulted in an increase of CXCL10 (Fig. 1g). miR-155^+^ILC2s positively correlated with IL-5 only in females (Fig. 1h) and negatively correlated with CXCL10 in all subjects (Fig. 1i). When evaluating CRTH2 and ST2 expression, we found increased numbers of miR-155^+^ILCs expressing ST2 specifically in females upon IL-33+PGD2 stimulation (Fig. 1j), suggesting that the increase in miR-155^+^ILC2s was a result of upregulated ST2 expression. Finally, we analyzed the CD4^+^Th cells and found similar miR-155 expression differences between the sexes as were seen in ILCs (Fig. 1k). These results show that the evaluation of receptor expression and miRNA expression can be performed by using the PrimeFlow™ RNA Assay method to analyze rare cells in a mixed-cell population. Due to the novelty of this method, only a limited number of subjects were recruited, and confirmation in a larger cohort would be valuable. Nevertheless, it is striking that even with our modest number of samples, a clear distinction between the sexes was found.

In conclusion, we report for the first time that human circulating ILCs express miR-155. Interestingly, female-derived ILCs and Th cells responded to various stimuli with higher miR-155 expression levels and numbers of miR-155-expressing cells than male-derived cells. miR-155 is a proinflammatory miRNA, and our data may contribute to the understanding of sex differences in inflammatory diseases such as asthma.

## Supplementary information

Supplementory information

## References

[1] Johansson K, Weidner J, Rådinger M (2018). MicroRNAs in type 2 immunity. Cancer Lett..

[2] Lai C, Stepniak D, Sias L, Funatake C (2018). A sensitive flow cytometric method for multi-parametric analysis of microRNA, messenger RNA and protein in single cells. Methods.

[3] Simoni Y, Newell EW (2018). Dissecting human ILC heterogeneity: more than just three subsets. Immunology.

[4] Johansson K, Malmhäll C, Ramos-Ramírez P, Rådinger M (2017). MicroRNA-155 is a critical regulator of type 2 innate lymphoid cells and IL-33 signaling in experimental models of allergic airway inflammation. J. Allergy Clin. Immunol..

[5] Malmhäll C (2014). MicroRNA-155 is essential for T(H)2-mediated allergen-induced eosinophilic inflammation in the lung. J. Allergy Clin. Immunol..

[6] Nwaru BI (2019). Cohort profile: the West Sweden Asthma Study (WSAS): a multidisciplinary population-based longitudinal study of asthma, allergy and respiratory conditions in adults. BMJ Open..

[7] Liew FY, Girard JP, Turnquist HR (2016). Interleukin-33 in health and disease. Nat. Rev. Immunol..

[8] Xue L (2014). Prostaglandin D2 activates group 2 innate lymphoid cells through chemoattractant receptor homologous molecule expressed on TH2 cells. J. Allergy Clin. Immunol..

[9] Florijn BW, Bijkerk R, van der Veer EP, van Zonneveld AJ (2018). Gender and cardiovascular disease: are sex-biased microRNA networks a driving force behind heart failure with preserved ejection fraction in women?. Cardiovasc Res..

[10] Bartemes K, Chen CC, Iijima K, Drake L, Kita H (2018). IL-33-Responsive Group 2 Innate Lymphoid Cells Are Regulated by Female Sex Hormones in the Uterus. J. Immunol..

