# Supplementary material for: MicroRNA-155 expression suggests a sex disparity in innate lymphoid cells at the single-cell level
Source: Cell Mol Immunol. 2019 Oct 10;17(5):544–6. doi: 10.1038/s41423-019-0303-4 (PMC7193614; doi:10.1038/s41423-019-0303-4)

**Supplementary Information**

**MicroRNA-155 expression suggests sex disparity in innate lymphoid cells at the single-cell level**

Carina Malmhäll, PhD; Julie Weidner, PhD; Madeleine Rådinger, PhD*

Krefting Research Centre, Sahlgrenska Academy, University of Gothenburg, Sweden

**Study subjects**

Study participants were recruited from the West Sweden Asthma Study cohort. In brief, asthmatics with physician diagnosed mild to moderate asthma and ongoing ICS treatment were included. Control subjects did not report asthma symptoms. Exclusion criteria was the common cold, other respiratory diseases, cancer or current smoking. The skin prick test was performed using a standard panel of 10 inhalant allergens composed of birch, mugwort, timothy, horse, dog, cat, *Cladosporium, Alternaria, Dematophagoides farina* and *D. pteronyssinus* (ALK, Horsholm, Denmark). All subjects performed spirometry and were measured for weight and height. Blood samples were collected and analyzed by the Clinical Chemistry Laboratory (Sahlgrenska University Hospital, Gothenburg, Sweden) to determine cellular composition by differential cell count and levels of C-reactive protein. Subjects with a CRP value above 5 were excluded. The study population consisted of 8 asthmatics and 5 non-asthmatic healthy controls, which was further divided based on sex resulting in 6 females and 7 males. All subjects gave written informed consent. Ethical approval for the study was granted by the Regional Ethical Approval Committee in Gothenburg, Sweden (no. 228-14)

**Table S1. Demographic and clinical characteristics of study subjects**

|  |  | **Divided based on clinical characteristics** | | **Divided based on sex** | |
| --- | --- | --- | --- | --- | --- |
| **Parameter** | **Units** | **Healthy** | **Asthmatics** | **Females** | **Males** |
| No. of subjects | {F/M} | 5 {2/3} | 8 {4/4} | 6 | 7 |
| Age | years | 48 (36-58) | 60 (27-67) | 48 (27-67) | 56 (46-67) |
| BMI |  | 24 (21-26) | 27.5 (21-31) | 26 (21-29) | 26 (24-31) |
| FEV1, predicted | % | 94.8 (83.7-116.2) | 91.4 (63.9-105.7) | 86 (73.5-105.7) | 93.8 (63.9-116.2) |
| FEV1/FVC | % | 82.5 (81-89) | 69.5 (58-86)* | 67 (58-86) | 75 (59-89) |
| C-reactive protein | mg/L | 0.5 (0.5-2) | 2 (0.5-4)* | 2 (0.5-4) | 1 (0.5-3) |
| B-Neutrophils | x10^9^/L | 3.2 (2.8-4) | 2.6 (2-4.3) | 3 (2.6-4.3) | 2.8 (2-4) |
| B-Lymphocytes | x10^9^/L | 1.8 (1.4-2.6) | 2 (1.4-2.9) | 2.05 (1.4-2.6) | 1.7 (1.4-2.9) |
| B-Monocytes | x10^9^/L | 0.4 (0.4-0.5) | 0.4 (0.3-0.8) | 0.4 (0.3-0.8) | 0.4 (0.3-0.5) |
| B-Eosinophils | x10^9^/L | 0.2 (0.08-0.2) | 0.25 (0.2-0.4)* | 0.25 (0.08-0.4) | 0.2 (0.2-0.3) |
| Skin prick test | +/- | 0/5 | 6/2 | 4/2 | 2/5 |
| Asthma (all on ICS) |  | 0 | 8 | 4 | 4 |

*P<.05 in comparison to healthy

Median values with range presented in ( )

BMI = Body Mass Index

FEV1 = Forced expiratory value in 1 second

FEV1/FVC = Forced expiratory value in 1 second/Forced vital capacity

ICS = Inhaled corticosteroids

**PrimeFlow™ RNA Assay**

PrimeFlow™ RNA assay was performed using a commercial assay kit (PrimeFlow™ RNA Assay, Invitrogen by Thermo Fisher Scientific, Affymetrix Inc., Santa Clara, California). All buffers mentioned below were provided in the Primeflow RNA assay Kit. PBMCs were processed throughout the staining and hybridization procedure according to manufacturer’s instructions (PrimeFlow™ RNA Assay with microRNA Pretreatment Protocol). Briefly described, PBMCs were incubated with 1mg/ml of Human IgG (Sigma, Saint Louis, Missouri) followed by staining with viability dye (Live/Dead™Fixable Aqua stain, Invitrogen, Life Technologies corp, Eugene, Oregon) and antibodies to detect surface antigens: Lin cocktail (CD3, CD14, CD16, CD19, CD20, CD56, CD123, CD11c, FcεRIα), ST2/IL-33R, CD127, CD4, CRTH2/CD294 (Table below). After the surface staining, PBMCs were treated with microRNA Pretreatment Buffer and thereafter fixed using Fixation buffer 1 before permeabilization with Permeabilization buffer containing RNase inhibitor. Samples were then fixed in Fixation buffer 2, washed and kept overnight at 4ᵒC in wash buffer with RNase inhibitor. To detect cellular miRNA, sequential hybridizations were performed in a dry incubator at 40ᵒC. Target probe sets were first hybridized, then signal amplification hybridization was carried out using pre-amplifierDNA and amplifierDNA followed by the corresponding fluorescent labeled probes (Type 1, Alexa Fluor 647; Type 4, Alexa Fluor 488). Probe sets used were the single pair target probe set for human miR-155-5p (MIMAT0000646, VM1-10254-PF, QuantiGene ViewRNA miRNA Probe Set, Affymetrix Inc.) and for each experiment a control probe, human ribosomal protein L13A (NM_012423, VA4-13187-PF, ViewRNA Probe, Affymetrix Inc.), was used in one tube to verify the hybridization process. Several steps of washing were performed throughout the staining and hybridization with appropriate buffers according to manufacturer’s protocol. Finally, in an additional step that was modified from manufacturer’s protocol, CD45-PerCP antibody was added and incubated for 10 min, cells were washed followed by cell analysis.

**Table S2. Antibodies for flow cytometric analysis**

| **Antigen (clone)** | **Format** | **Manufacturer** |
| --- | --- | --- |
| Lineage cocktail:CD3 (SK7), CD14 (MΦP9), CD16 (3G8), CD19 (SJ25C1), CD20 (L27), CD56 (NCAM16.2) | FITC | BD Biosciences, San Jose, California |
| CD123 (7G3) | FITC | BD Pharmingen™, BD Biosciences |
| CD11c (B-ly6) | FITC | BD Pharmingen™, BD Biosciences |
| FcεRI (AER-37) | FITC | BioLegend, San Diego, California |
| ST2/IL-33R (polyclonal Goat IgG) | PE | R&D Systems®Minneapolis, Minnesota |
| CD45 (2D1) | PerCP | BD Biosciences |
| CD127 (HIL-7R-M21) | Pe-Cy™7 | BD Pharmingen™, BD Biosciences |
| CD4 (RPA-T4) | APC-H7 | BD Pharmingen™, BD Biosciences |
| CRTH2/CD294 (BM16) | BV421 | BD Horizon™, BD Biosciences |

APC, Allophycocyanin;APC-H7, H7 conjugate of Allophycocyanin; BV421, Brilliant Violet 421; FITC, Fluorescein isothiocyanate; PE, phycoerythrin; PE-Cy7, Cy7 conjugate of phycoerythrin; PerCP, Peridinin chlorophyll protein.

**Flow cytometry**

All flow cytometric analyses were performed with a BD FACSVerse Flow Cytometer running BD FACSuite Software (BDBiosciences, San Jose, California). Mean number of acquired cells was 1.02 x 10^6^ cells. Data were analyzed with FlowJo Software (Tree Star Inc., Ashland, Oregon). Only live singlets CD45+ lymphocytes were analyzed. Lineage negative cells were determined as CD3-, CD14-, CD16-, CD19-, CD20-, CD56-, CD123-, CD11c- and FcεRI-. ILCs were determined as Lin- and CD127+. ILCs positive for either CRTH2 or ST2 or the combination were considered ILC2s. Lin+ CD4+ cells were considered Th cells. Gating of miR-155 and surface markers were determined using control samples by the Fluorescence minus one (FMO) approach i.e. controls containing all markers except the one of interest were used to set gates. Gating strategy can be seen below.

**Figure S1. Gating strategy**


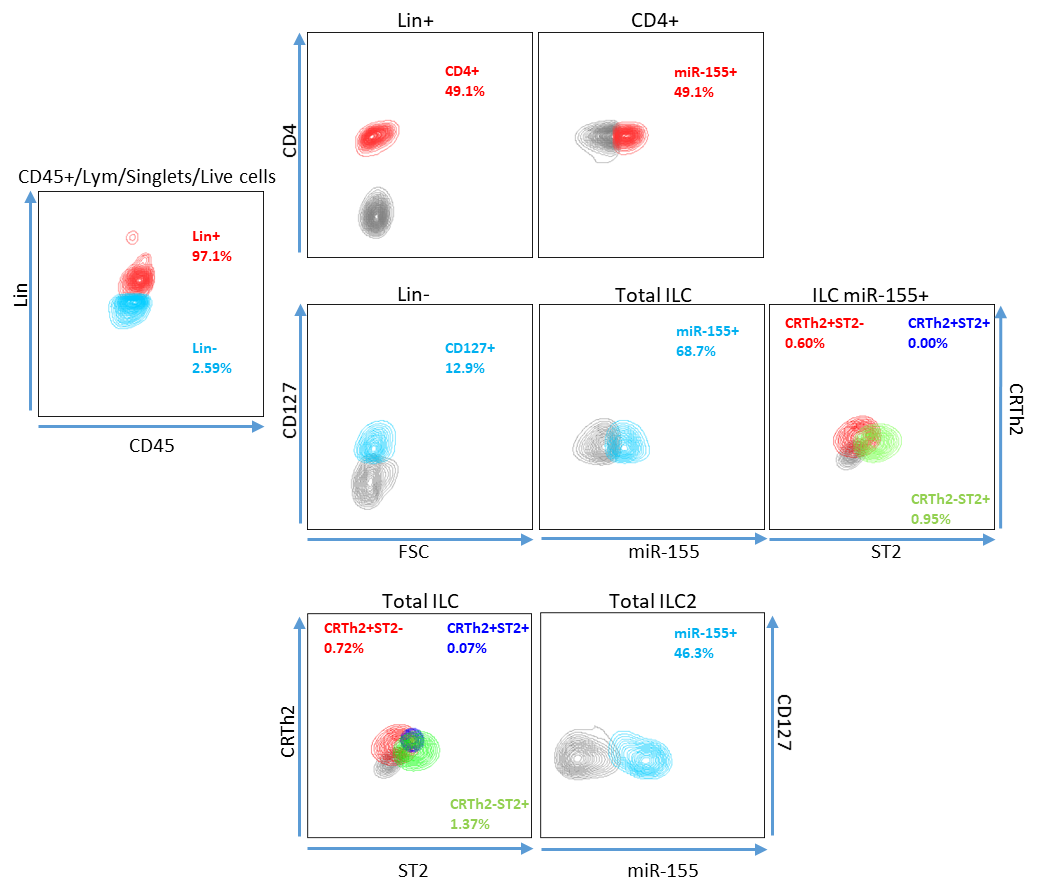

Supplement: Supplementary file 1 — Supplementory information [file 41423_2019_303_MOESM1_ESM.docx]
